# Pharmacotherapeutic Advancements in Personalized Medicine: A Comprehensive Review of Emerging Trends in Clinical Pharmacy Practice

**DOI:** 10.7759/cureus.108329

**Published:** 2026-05-05

**Authors:** Rajkumari Bansal, Helen William, Mohit Kher, Yagnik Prafulchandra Tank, Bansari Yagnik Tank, Ashutosh Shukla

**Affiliations:** 1 School of Medical Science, Department of Pharmacology, Sri Satya Sai University and Technology, Pachama, IND; 2 Department of Pharmaceutical Sciences, Government Medical College, Kottayam, Kottayam, IND; 3 Department of Pharmacology, Kanti Devi University, Kanti Devi Medical College Hospital and Research Centre, Mathura, IND; 4 Department of Pathology, Dr. N. D. Desai Faculty of Medical Science and Research, Dharmsinh Desai University, Nadiad, IND; 5 Department of Microbiology, Dr. N. D. Desai Faculty of Medical Science and Research, Dharmsinh Desai University, Nadiad, IND; 6 Department of Life and Health Sciences, Indus University, Ahmedabad, IND

**Keywords:** biomarker-guided therapy, clinical pharmacy practice, model-informed dosing, personalised pharmacotherapy, pharmacogenomics integration

## Abstract

Pharmacotherapeutic advancements in personalised medicine, clinical pharmacy practice, pharmacogenomics (PGx), model-informed precision dosing (MIPD), therapeutic drug monitoring (TDM), and artificial intelligence (AI) are increasingly shaping individualised drug therapy. Personalised pharmacotherapy offers substantial potential to improve safety and effectiveness, yet translation into routine care remains inconsistent across disease areas and health systems. Gaps persist in integrating biological insight, quantitative tools, and workflow-ready decision support into coherent therapeutic strategies. This review aimed to critically evaluate emerging pharmacotherapeutic trends and their practical integration within clinical pharmacy practice. A comprehensive review methodology was employed, drawing evidence from PubMed, Scopus, Web of Science, and Google Scholar, with literature published between 2015 and 2025 prioritised. Conceptual integration and comparative analysis were applied to peer-reviewed clinical and modelling studies relevant to pharmacotherapeutic decision-making. The review highlights that personalisation achieves greatest value when PGx, biomarkers, MIPD, and digital tools are applied as interconnected components rather than isolated interventions. Evidence indicates improved therapeutic alignment, reduced variability, and enhanced decision consistency in complex care settings when integrative approaches are adopted. These findings underscore the importance of structured implementation and continuous reassessment to support individualised therapy. Long-term implications include expanded roles for data-enabled medication management and improved scalability of precision approaches across healthcare systems and clinical environments.

## Introduction and background

The idea of personalised medicine has evolved into a paradigm shift in contemporary healthcare, challenging the traditional one-size-fits-all approach to pharmacotherapy and reshaping therapeutic decision-making in heterogeneous patient populations [1]. Advances in molecular biology, genomics, and clinical informatics have revealed substantial interindividual variability in drug response, efficacy, and toxicity, necessitating patient-specific treatment strategies that extend beyond conventional dosing algorithms [2]. Clinical pharmacy practice is positioned centrally within this evolving landscape, linking biological and computational insights to actionable pharmacotherapeutic decisions at the point of care [3]. Personalised pharmacotherapy is grounded in the recognition that drug response is influenced by a dynamic interplay of genetic, physiological, environmental, and behavioural factors [4]. Although early conceptualisation emphasised improved outcomes and reduced adverse events, translation into routine clinical practice has remained inconsistent and context-dependent [5]. Over the past decade, the field has progressed toward partial clinical implementation through pharmacogenomics (PGx), biomarker-guided therapy, and model-informed dosing approaches that support therapeutic optimisation [6].

The integration of pharmacogenomic data into medication management and decision-support systems has expanded the role of clinical pharmacists to include interpretation of genetic information, prediction of drug-gene and drug-drug interactions, and delivery of individualised therapy across complex clinical domains such as psychiatry, cardiovascular disease, oncology, and critical care [7]. Concurrent advances in computational sciences, including machine learning and pharmacokinetic/pharmacodynamic modelling, have enabled adaptive dosing and predictive analytics based on patient-specific data streams [8]. However, the clinical impact of these innovations remains uneven, with limited evidence demonstrating consistent improvements in real-world outcomes across diverse healthcare settings [9]. This gap reflects not only biological complexity but also challenges related to evidence quality, workflow integration, clinician readiness, and inequitable access to precision technologies [5]. Additional constraints, including cost-effectiveness concerns and infrastructure limitations, further hinder large-scale implementation [10].

Genetic information alone is often insufficient to predict therapeutic response due to modifying factors such as phenoconversion, comorbidities, inflammation, and polypharmacy, which can alter expected genotype-phenotype relationships [11]. These limitations have driven the development of integrative models combining clinical pharmacy practice, PGx, pharmacokinetics, pharmacodynamics, therapeutic drug monitoring (TDM), and real-time clinical data [12]. Such approaches necessitate a transition from static, test-based decision-making to adaptive, learning-based pharmacotherapy models within clinical pharmacy practice [9]. While personalised strategies have demonstrated benefits in selected patient populations and disease conditions, their predictive reliability and generalisability remain variable, limiting broader applicability [7]. These observations highlight the need for critical evaluation of emerging trends, not only in terms of technological innovation but also regarding clinical utility, patient-centred outcomes, and system-level impact [6].

Despite substantial advancements, a clear knowledge gap persists in understanding how these diverse precision tools can be systematically integrated into coherent, workflow-ready pharmacotherapeutic strategies that consistently improve clinical outcomes. Current literature often examines PGx, biomarkers, modelling, and digital tools in isolation, with limited synthesis of their combined impact on medication management and healthcare value [3]. Moreover, insufficient attention has been given to balancing innovation with practical constraints, including variability in evidence strength and implementation feasibility [5].

Accordingly, this review aims to provide an integrated and critical synthesis of emerging pharmacotherapeutic approaches in personalised medicine, with particular emphasis on their convergence within clinical pharmacy practice. It seeks to clarify the translational challenges, identify areas of inconsistency, and evaluate the extent to which these innovations can support structured, outcome-oriented, and scalable personalised pharmacotherapy.

Objectives of the review

This review critically evaluates key advances in personalised pharmacotherapy, focusing on PGx, biomarker-guided therapy, model-informed precision dosing (MIPD), and digital decision-support tools, and assesses their integration and applicability within clinical pharmacy practice.

Methodology

Study Design

This study was conducted as a comprehensive narrative review employing a structured literature search and thematic synthesis approach, without adherence to a formal systematic review framework such as PRISMA. The methodology emphasises conceptual synthesis and comparative analysis of mechanistic, clinical, and implementation-oriented studies rather than quantitative meta-analysis. This review does not follow a formal systematic review or PRISMA protocol, as its objective is to provide a comprehensive, integrative, and critical synthesis of emerging evidence rather than a quantitative or strictly protocol-driven analysis. Accordingly, a structured narrative approach was adopted to allow flexibility in integrating mechanistic, clinical, and implementation-oriented evidence across diverse domains.

Information Sources

A structured literature search was conducted across PubMed, Scopus, Web of Science, and Google Scholar to capture relevant pharmacological, genomic, and health systems research.

Search Strategy

Both controlled vocabulary and free-text terms were applied, including keywords related to precision medicine, personalised pharmacotherapy, PGx, biomarker-guided therapy, MIPD, artificial intelligence (AI), and clinical pharmacy practice. The search was restricted to publications from 2015 to 2025 to ensure inclusion of recent scientific advances while retaining relevant foundational studies where necessary.

The search strategy incorporated Boolean operators (AND, OR) to combine search terms and database-specific controlled vocabulary (e.g., MeSH in PubMed). Example search strings included: (“precision medicine” OR “personalised pharmacotherapy”) AND (“pharmacogenomics” OR “biomarkers”) AND (“model-informed dosing” OR “artificial intelligence” OR “clinical pharmacy”). Database-specific filters, including language and study type, were applied where appropriate.

Study Selection

Eligible studies included peer-reviewed original research and methodologically robust modelling studies with direct relevance to pharmacotherapeutic decision-making. Studies limited to preclinical investigations or lacking translational or clinical applicability were excluded.

Study selection was conducted through a staged screening process, including title and abstract screening followed by full-text evaluation. Studies were assessed for relevance based on predefined criteria. Where necessary, discrepancies in study selection were resolved through consensus-based discussion.

Inclusion and Exclusion Criteria

Studies were included if they were peer-reviewed clinical or modelling-based investigations addressing personalised pharmacotherapy, including PGx, biomarker-guided therapy, MIPD, digital health technologies, or clinical pharmacy practice, with direct clinical or translational relevance, and published between 2015 and 2025. Studies were excluded if they were limited to preclinical or in vitro research without clinical applicability, lacked relevance to pharmacotherapeutic decision-making, or were non-peer-reviewed or methodologically weak sources.

Data Evaluation and Extraction

Selected sources were evaluated based on clinical relevance, applicability to practice, and implementation considerations. Key concepts, methodological approaches, and clinical implications were extracted to support comparative analysis.

Quality Appraisal

Given the narrative nature of this review, formal risk-of-bias assessment tools (e.g., Preferred Reporting Items for Systematic Reviews and Meta-Analyses (PRISMA), A MeaSurement Tool to Assess Systematic Reviews (AMSTAR), and Risk Of Bias In Non-randomized Studies of Interventions (ROBINS-I)) were not applied. However, the included studies were critically appraised based on methodological robustness, clinical relevance, and applicability to practice to ensure inclusion of high-quality and contextually meaningful evidence.

Data Synthesis

The included studies were thematically organised to facilitate integrative analysis across emerging trends in personalised pharmacotherapy, with emphasis on PGx, biomarker-guided therapy, model-informed dosing, and digital health integration.

Themes were derived through iterative comparison of study objectives, methodologies, and outcomes. Comparative analysis was applied to identify converging evidence, inconsistencies, and translational challenges across domains. Greater interpretive emphasis was placed on clinically validated and implementation-focused studies.

Quantitative synthesis methods such as meta-analysis or meta-regression were not performed due to substantial heterogeneity across included studies in terms of design, populations, interventions, and reported outcomes. Accordingly, a structured narrative synthesis approach was adopted to enable comparative interpretation of findings across mechanistic, clinical, and implementation domains. Evidence was evaluated qualitatively, with greater interpretive emphasis placed on studies demonstrating clinical validation, methodological robustness, and real-world applicability. This approach allowed the identification of converging trends, inconsistencies, and translational gaps without imposing inappropriate statistical aggregation

## Review

Evolution of personalised pharmacotherapy in clinical pharmacy

Paradigm Shift in Pharmacotherapy Practice

Personalised pharmacotherapy has evolved from a theoretical concept into a practical clinical paradigm, driven by the recognised limitations of population-based prescribing in routine care [1]. Exploration of interindividual variability in drug exposure and response has highlighted its impact on efficacy and safety, particularly in chronic and high-risk patient populations [13]. Clinical pharmacy has played a central role in translating this understanding into practice by redefining medication management as patient-centred rather than strictly guideline-driven [2]. This shift reflects a departure from reliance on average pharmacokinetic models toward recognition of multilevel determinants of drug behaviour, including genetic variability, disease-related physiological changes, and environmental influences [14]. These determinants increasingly inform therapeutic decision-making through tools such as PGx interpretation, TDM, and exposure-response assessment, enabling more context-specific prescribing [3]. Consequently, pharmacotherapy has progressed from associative evidence toward actionable, decision-oriented frameworks that support individualised care [4].

Evolution of Clinical Pharmacy Practice

**Figure 1:**
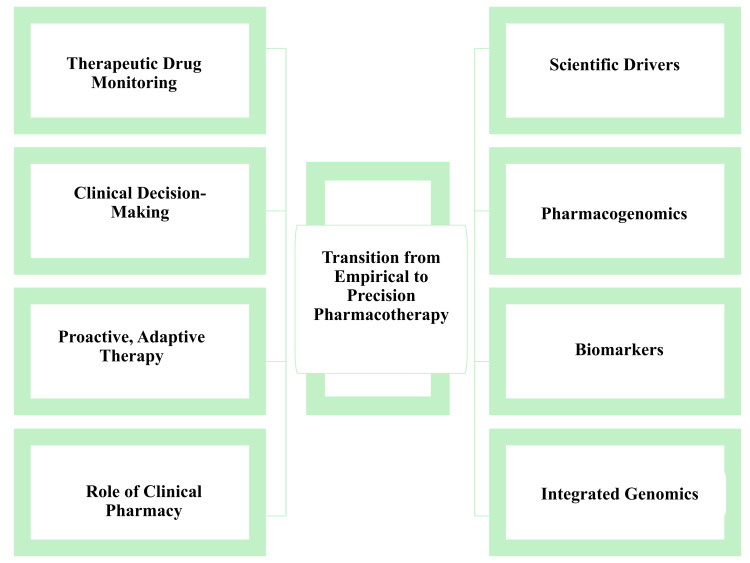
Transition from conventional dosing to precision pharmacotherapy Created by the authors using Microsoft PowerPoint (Microsoft Corp., Redmond, WA, USA).

The advancement of individualised pharmacotherapy has been shaped by the integration of mechanistic modelling, real-world evidence, and clinical decision-support systems [15]. Contemporary approaches emphasise continuous assessment and adaptive adjustment of therapy, rather than viewing personalisation as a one-time intervention [5]. Clinical pharmacy practice has correspondingly expanded beyond traditional dose verification to encompass stewardship of personalised therapeutic regimens, particularly in the context of polypharmacy and physiological instability [6]. This role is especially critical in clinical scenarios with narrow therapeutic margins, where dosing inaccuracies can have significant consequences and require integration of complex patient-specific data at the point of care [16]. Current evidence supports the view that individualised pharmacotherapy represents a dynamic and integrative model of clinical practice, rather than a collection of isolated technological interventions [7]. Figure 1 illustrates the transition from conventional dosing approaches to precision pharmacotherapy.

Determinants of Interindividual Variability in Drug Response

The problem of interindividual differences in drug response is among the most significant to successful pharmacotherapy and is the scientific rationale why individualised practices are significant to the clinical pharmacy practice [8]. The variability is achieved by the interplay of genetic determinants, physiological states, exposures to environmental factors, and treatment-related factors, which give rise to patterns of response that cannot be predicted accurately using purely population means [9]. Pharmacodynamic heterogeneity at the target drug and signalling pathways level caused by the difference in absorption, distribution, metabolism, and elimination is likely to be overlapping with the pharmacodynamic heterogeneity of the drug targets and drug-signalling, which increases the unpredictability in clinical outcome [10].

Genetic variations in the enzymes and transporters of drugs represent differences in drug metabolism, which have a role in the differences in exposures, but the genotype does not often play a role in the observed therapeutic responses [11]. The most typical examples of phenotypic modifiers include inflammation, organ dysfunction, age, and comorbid disease that alter enzyme activity and receptor sensitivity in real-life scenarios, thereby leading to genotype-phenotype discordance [12]. This effect is the cause of the need to integrate the fixed genetic data with the dynamically shifting clinical markers in the process of individualising therapy [17]. In order to continue with the personalisation, the models that can model multidimensional variability rather than single predictor separation are required [1]. Pharmacodynamics and pharmacokinetic modelling and TDM may provide the quantitative forms of contextualization of the patient-specific data, and may be used to modify the dose. According to the changing physiology or treatment conditions [13]. Such tools may prove to be most beneficial within properly organised clinical procedures, which enable the reconsideration to be repeated rather than being done at a few instances [14].

Recognition of factors of variability makes it possible to stratify risks, monitor the severity of the risk, and select the strategies of personalisation in the sphere of treatment [18]. This kind of knowledge is put into practice by forecasting the outcomes of interaction, settling conflicting sources of variability, and creating therapeutic recommendations on the basis of mechanistic understanding into a pragmatic form [2]. The interindividual variability is one of the key aspects of the accuracy of pharmacotherapy, well-known, and it is the interaction of biological variability with the complex clinical environment [19]. Variability has therefore been dealt with as a common theme that informs the decision-making in individualised therapeutic strategies that inform literature on pharmacy-related practice [20]. Table 1 contains the most important points in the differences in interindividual drug response.

**Table 1:** Determinants of interindividual variability in drug response PK: pharmacokinetics; PD: pharmacodynamics; CYP: cytochrome P450; DDI: drug–drug interaction

Determinant Category	Key Contributing Factors	Mechanistic Influence on Drug Response	Implications for Personalised Pharmacotherapy	References
Genetic variability	Polymorphisms in CYP enzymes, transporters, and drug targets	Alters metabolism, clearance, receptor binding, and signalling	Supports genotype-informed drug selection and dosing, but is insufficient as a standalone predictor	[20]
Physiological state	Age, renal/hepatic function, inflammation, pregnancy	Modifies enzyme activity, protein binding, and distribution	Necessitates phenotype-informed dose adjustment and monitoring	[10]
Disease-related factors	Comorbidities, disease severity, and acute illness	Alters PK/PD relationships and target responsiveness	Reinforces the need for dynamic, context-aware personalisation	[1]
DDI	Enzyme inhibition/induction, transporter competition	Causes phenoconversion and unpredictable exposure changes	Requires proactive interaction assessment and therapy modification	[18]
Behavioural & environmental factors	Adherence, diet, smoking, supplements	Influences exposure consistency and therapeutic reliability	Highlights the importance of pharmacist-led medication reconciliation	[3]

PGx as a foundation for precision pharmacotherapy

Principles and Mechanistic Basis

PGx has emerged as a foundational component of personalised pharmacotherapy by providing a molecular framework to anticipate variability in drug response and the risk of adverse outcomes [3]. It extends beyond genetic testing alone to encompass the interpretation of genomic data within complex therapeutic decision-making processes [21]. Genetic variants affecting drug-metabolising enzymes, transporters, and pharmacological targets offer mechanistic insights into drug exposure and response, and must be integrated with pharmacokinetic, pharmacodynamic, and clinical parameters for meaningful application [11]. These advances have shifted PGx from association-driven discovery toward actionability-oriented, implementation-focused models in clinical practice [22]. Increasingly, pharmacist-led models incorporate genotype-based recommendations into prescribing workflows, enabling early identification of high-risk therapies and alternative treatment strategies [1]. However, variability in effect size across drugs and populations indicates that PGx is most appropriately used as a stratification tool rather than a definitive predictor of therapeutic outcomes [12].

Clinical Applications and Utility

In clinical practice, PGx contributes to optimising therapy selection, particularly in settings characterised by narrow therapeutic indices, cumulative toxicity, or significant interpatient variability [23]. Genotype-informed decision-making can enhance the efficiency of prescribing by reducing reliance on trial-and-error approaches. The clinical utility of PGx is further supported by its role in contextualising patient-specific data, facilitating communication between clinicians and patients, and aligning therapeutic choices with safety considerations [6]. Evidence suggests that the greatest benefit is achieved when PGx is integrated within broader pharmacotherapeutic frameworks, combining genomic insights with adaptive clinical evaluation and monitoring [24]. At the system level, such integration has been associated with improved consistency, accountability, and scalability of genomics-informed decision-making in healthcare settings [5].

Limitations and Implementation Challenges

Despite its potential, the clinical application of PGx is constrained by several limitations that affect its predictive reliability and implementation. Phenoconversion, polypharmacy, and disease-related alterations in enzyme activity can modify or override genotype-based predictions, leading to discordance between expected and observed drug responses [17]. These challenges necessitate dynamic assessment strategies that incorporate both genomic data and real-time clinical variables, reinforcing the need for interdisciplinary collaboration and continuous evaluation [13]. Accordingly, PGx is most effective when positioned as one component within a multivariate decision-making framework rather than as a standalone solution [4]. The complexity of integrating PGx into routine care also reflects broader issues related to clinical interpretation, infrastructure, and workflow adaptation, highlighting the importance of structured implementation and ongoing evaluation to ensure safe and effective use.

Biomarker-guided therapeutic selection and monitoring

Types and Functional Roles of Biomarkers

Biomarker-based pharmacotherapy represents a key component of personalised medicine by linking quantitative biological measurements with therapeutic decision-making in clinical practice [7]. Unlike pharmacogenomic markers, which are largely based on fixed genetic predispositions, biomarkers reflect dynamic changes in disease activity, drug exposure, and treatment response, thereby providing real-time insights into therapeutic effectiveness [25]. This dynamic nature makes biomarkers versatile tools for guiding treatment selection, dose adjustment, and therapeutic monitoring across diverse clinical settings [9]. Biomarkers function across a spectrum that includes predictive indicators of treatment response, pharmacodynamic markers of target engagement, and measures of toxicity risk [10]. Their clinical value lies not only in their association with outcomes but also in their ability to inform and modify therapeutic decision-making to improve patient care [26]. However, their application requires robust validation frameworks to distinguish clinically actionable biomarkers from those that merely reflect disease pathways or surrogate endpoints [11].

Clinical Applications in Therapy Selection and Monitoring

In clinical practice, biomarker-guided strategies are most effective when integrated with clinical judgment, pharmacokinetic evidence, and patient-centred considerations [12]. Increasing emphasis is placed on demonstrating biomarker performance under real-world conditions, accounting for variability in assay reliability, timing of measurement, and threshold definitions [17]. Incorporating biomarker data into structured medication management plans and predefined monitoring protocols enhances their utility in guiding therapeutic adjustments [3]. Biomarker-guided approaches are particularly valuable in conditions characterised by heterogeneous treatment responses or narrow therapeutic indices, where they can optimise therapy and reduce unnecessary exposure to ineffective treatments [27]. Such integration supports more precise and responsive pharmacotherapy by enabling iterative evaluation of treatment effectiveness.

Limitations and Validation Challenges

**Figure 2:**
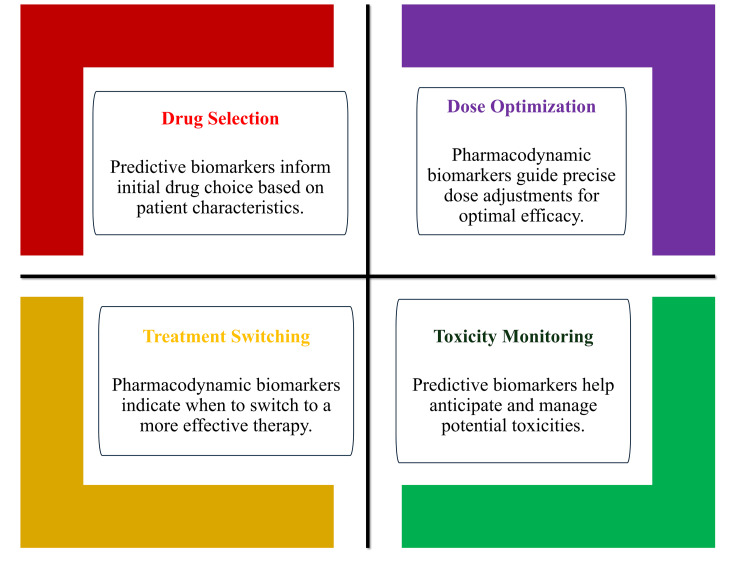
Use of biomarkers to guide individualised therapy selection and monitoring Created by the authors using Microsoft PowerPoint (Microsoft Corp., Redmond, WA, USA).

Despite their potential, the clinical impact of biomarkers remains inconsistent due to challenges in standardisation, validation, and translation into routine practice. Variability in assay performance, lack of universally accepted thresholds, and differences in clinical interpretation can limit reliability and comparability across settings [18]. These limitations highlight the need for structured implementation pathways and rigorous performance evaluation frameworks. Furthermore, overreliance on isolated biomarker measurements without contextual clinical interpretation may lead to suboptimal decision-making, reinforcing the importance of integrated models that combine mechanistic understanding with clinical judgment [4]. The role of biomarkers in personalised pharmacotherapy, therefore, remains context-dependent, requiring continuous reassessment and alignment with evolving clinical evidence [28]. Figure 2 illustrates the role of biomarkers in guiding individualised therapy selection and monitoring.

Model-informed precision dosing and therapeutic drug monitoring

Concept and Modelling Framework

MIPD represents a quantitative approach to operationalising personalised pharmacotherapy by integrating patient-specific data into dose optimisation strategies. This approach combines pharmacokinetic and pharmacodynamic principles with population modelling and Bayesian forecasting to determine optimal dosing for individual patients [29]. Compared to conventional fixed-dose adjustments, model-informed strategies can account for dynamic physiological changes, organ function variability, and treatment context, enabling adaptive therapy over time [13]. Precision dosing frameworks incorporate multiple covariates, including body weight, renal function, genotype, disease state, and concomitant medications, to explain variability in drug disposition and response [10]. TDM serves as a critical feedback mechanism within this framework, validating model predictions through measured drug concentrations and informing subsequent dose adjustments [30]. The integration of modelling and monitoring allows clinicians to balance proactive and reactive strategies in controlling drug exposure [1].

Clinical Applications Across Therapeutic Areas

MIPD has demonstrated particular utility in clinical settings characterised by high variability and narrow therapeutic indices, such as critical care, transplant medicine, and oncology. Pharmacist-guided implementation of model-informed dosing has been associated with improved target attainment and reduced toxicity in complex patient populations [31]. The approach enhances therapeutic precision by enabling continuous dose optimisation based on evolving patient conditions, rather than relying on static dosing strategies. The application of MIPD reflects a broader shift toward data-driven pharmacotherapy, supporting sustained optimisation of treatment outcomes across diverse clinical environments [4]. Table 2 summarises key applications of MIPD across therapeutic domains.

**Table 2:** Applications of model-informed precision dosing across therapeutic areas TDM: therapeutic drug monitoring; PK: pharmacokinetics; PD: pharmacodynamics; TI: Therapeutic Index; ICU: intensive care unit

Therapeutic Area	Drugs Commonly Applied	Modelling & Monitoring Strategy	Clinical Benefit	Practice Considerations	References
Critical care	Aminoglycosides, vancomycin, beta-lactams	Bayesian PK models with TDM feedback	Improved target attainment, reduced toxicity	Requires rapid sampling, informatics support	[32]
Transplant medicine	Tacrolimus, cyclosporine	Population PK with covariate adjustment	Reduced rejection and nephrotoxicity	High interpatient variability, narrow TI	[18]
Oncology	Methotrexate, targeted agents	Exposure–response modelling	Optimised efficacy with toxicity control	Limited sampling feasibility	[30]
Infectious diseases	Antifungals, antivirals	Adaptive dosing using real-time concentrations	Improved outcomes in resistant infections	Assay availability and turnaround time	[3]
Special populations	Paediatrics, geriatrics, and ICU patients	Individualised covariate-rich models	Reduced dosing errors	Requires specialised pharmacist expertise	[12]

Operational and Implementation Considerations

Successful implementation of MIPD requires models that are transparent, clinically interpretable, and seamlessly integrated into healthcare workflows [17]. Adoption is facilitated by timely access to patient data, appropriate technological infrastructure, and targeted training for healthcare professionals [6]. The expanding role of clinical pharmacists in this context includes interpretation of model outputs, contextualisation of dosing recommendations, and balancing competing clinical risks during therapy adjustment [3]. These responsibilities necessitate expertise in pharmacometrics and clinical decision-making, as well as collaboration within multidisciplinary teams [12]. Despite its advantages, MIPD introduces challenges related to workflow integration, reliance on data quality, and potential overdependence on model-driven decisions, requiring structured governance and continuous evaluation [18]. The effective translation of quantitative modelling into routine clinical practice depends on robust monitoring systems and well-defined care processes [32]. Table 2 gives the application of model-informed precision dosing within different fields of therapy.

Advanced therapeutics and individualised pharmacotherapy

Mechanistic Complexity and Therapeutic Characteristics

Advanced therapeutics have intensified the need for personalised pharmacotherapy due to their reliance on complex biological mechanisms rather than linear dose-response relationships [33]. Biologics, monoclonal antibodies, and cell- and gene-based therapies exhibit features such as target-mediated drug disposition, nonlinear clearance, and immunogenicity, which challenge traditional dosing paradigms in clinical pharmacy [14]. These characteristics necessitate integration of molecular target interactions, disease activity, and patient-specific immune responses to accurately interpret drug exposure and therapeutic effect [7]. Consequently, personalised strategies for advanced therapeutics rely on the combined use of pharmacokinetic modelling, biomarker monitoring, and selective pharmacogenomic insights to guide therapy initiation, adjustment, and discontinuation [29]. TDM has become particularly relevant in this context to differentiate pharmacokinetic failure from pharmacodynamic resistance, thereby enabling more precise treatment modifications [34]. Variability in assay performance, exposure-response relationships, and clinical endpoints further complicates interpretation and requires structured evaluation frameworks [12].

Clinical Applications and Stewardship

The application of personalised approaches in advanced therapeutics is particularly critical in chronic inflammatory and oncologic conditions, where treatment courses are prolonged, complex, and resource-intensive [6]. In these settings, pharmacotherapy extends beyond dose optimisation to include stewardship functions such as management of immunogenicity, adherence monitoring, and coordination of multidisciplinary decision-making [35]. This expanded role reflects a shift in clinical pharmacy practice from traditional medication management toward comprehensive therapy systems management [1]. Failure to optimise dosing or delayed therapeutic adjustments can result in treatment resistance, toxicity, or unnecessary escalation of therapy, highlighting the clinical and economic importance of timely intervention [18]. Personalised strategies supported by modelling and monitoring can improve durability of response and enhance resource utilisation [30].

Implementation Challenges and System Requirements

Despite their potential, the integration of advanced therapeutics into personalised pharmacotherapy is constrained by significant implementation challenges. These include the need for specialised infrastructure, access to high-quality data, and advanced practitioner expertise to support complex decision-making processes [31]. The convergence of biological complexity and clinical requirements necessitates robust systems capable of integrating mechanistic data with real-time clinical information. Advanced therapeutics, therefore, represent a critical intersection of innovation and clinical application, reinforcing the need for structured frameworks to support their safe, effective, and scalable use in personalised pharmacotherapy [36].

Digital health, AI, and decision support tools

Data Integration and Predictive Capabilities

Digital health technologies and AI are increasingly central to personalised pharmacotherapy by enabling continuous data capture, predictive modelling, and real-time clinical decision-making [37]. Machine learning approaches can model complex, nonlinear relationships between patient characteristics, drug exposure, and clinical outcomes, thereby supporting optimised therapeutic decisions in dynamic care environments compared to conventional rule-based systems [15]. In clinical pharmacy practice, the value of these tools lies not solely in automation but in their ability to enhance clinical interpretation and support context-specific medication management [3]. AI-based systems can integrate diverse data sources, including electronic health records, laboratory results, pharmacokinetic data, and genomic or biomarker information [21]. This integration enables identification of latent patterns not readily detectable through traditional analytical methods, thereby improving risk prediction and dose individualisation [38]. However, the reliability of such models depends on data quality, representativeness, and transparency of model development, as poorly specified systems may introduce bias or generate clinically unreliable outputs [12].

Clinical Utility and Decision Support

The clinical utility of AI-driven decision-support tools depends on prospective validation and adherence to established pharmacological principles [29]. Effective implementation requires pharmacist oversight to ensure interpretability, appropriateness, and safe application of algorithm-based recommendations, particularly in settings involving narrow therapeutic indices or competing clinical priorities [6]. Systems designed to complement rather than replace professional judgment through collaborative interfaces are more likely to support iterative evaluation and continuous learning [1]. These tools demonstrate particular relevance in chronic disease management and complex inpatient settings, where real-time monitoring and adaptive adjustments can enhance therapeutic consistency and efficiency [39]. Digital platforms also facilitate scalability by standardising decision processes across institutions while maintaining patient-level individualisation [18].

Limitations, Bias, and Implementation Challenges

**Figure 3:**
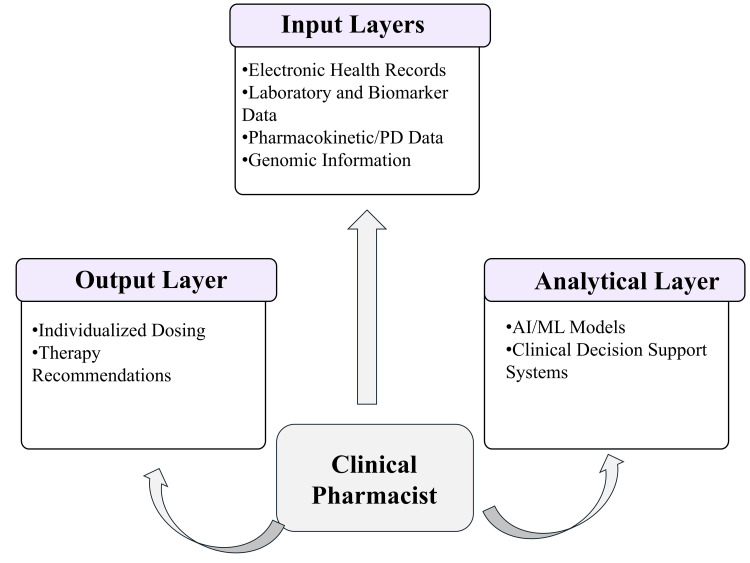
Role of digital tools in personalised pharmacotherapy PD: pharmacodynamics; AI: artificial intelligence; ML: machine learning Created by the authors using Microsoft PowerPoint (Microsoft Corp., Redmond, WA, USA).

Despite their potential, AI-driven tools present several challenges related to implementation, validation, and clinical integration. Their effectiveness depends on robust workflow design, user training, and governance frameworks to ensure accountability and consistent application in practice [40]. Limitations such as algorithmic bias, lack of transparency, and variability in data quality may affect reliability and clinician trust, underscoring the need for continuous evaluation and regulatory oversight. Accordingly, successful integration requires balancing technological innovation with clinical judgment and system-level readiness. Figure 3 illustrates the role of digital tools and pharmacist-supported decision-making in personalised pharmacotherapy.

Comparative Analysis

While PGx, biomarker-guided therapy, MIPD, and AI-driven decision-support tools all contribute to personalised pharmacotherapy, they differ in scope, application, and limitations. PGx primarily provides a static, genotype-based framework for anticipating drug response and guiding initial therapy selection, but its predictive accuracy may be limited by phenoconversion and clinical variability. In contrast, biomarkers offer dynamic, real-time insights into disease activity and treatment response, enabling ongoing monitoring and therapy adjustment, although their clinical utility depends on validation and standardisation. MIPD integrates pharmacokinetic and pharmacodynamic modelling with patient-specific variables to optimise dosing quantitatively over time, offering high precision in drugs with narrow therapeutic indices, yet requiring specialised expertise and infrastructure. AI and digital tools extend these approaches by integrating multidimensional data and enabling adaptive, real-time decision-making; however, their reliability is contingent on data quality, model transparency, and clinical validation. Collectively, these approaches are most effective when applied in a complementary manner, where PGx informs baseline decisions, biomarkers and TDM enable dynamic monitoring, MIPD refines dosing, and AI facilitates integrative, data-driven optimisation within clinical workflows.

Integration into clinical pharmacy practice and health systems

Workflow Integration and System Design

The transition towards individualised pharmacotherapy as a routine component of clinical practice requires harmonisation between scientific advancements and healthcare system infrastructure [41]. Although the technical foundation comprises genomic, biomarker, modelling, and digital technologies alongside clinical individualisation strategies, these elements must be effectively integrated into clinical workflows to enable timely interpretation and implementation of therapeutic decisions [3]. This gap can be addressed through integrative roles that translate complex biological and clinical data into actionable pharmacotherapeutic strategies in real-world care settings [6]. Redesigning medication-use processes to support continuous and iterative assessment, rather than episodic decision-making, is essential for successful integration [18]. This includes embedding decision-support outputs within prescribing and monitoring pathways, clearly defining pharmacist roles in interpretation and follow-up, and ensuring interoperability between laboratory, clinical, and pharmacy information systems [29]. Evidence-based implementation models further indicate that individualisation strategies must be adaptable to local resources, patient populations, and care delivery models to achieve sustainable impact [12].

Workforce Development and Interdisciplinary Collaboration

Education and competency development are critical enablers of integration, requiring pharmacists to develop expertise in PGx, pharmacometrics, and data-driven decision-making [31]. Interdisciplinary collaboration is increasingly important, with pharmacists working alongside physicians, data scientists, and laboratory specialists in therapeutic planning [1]. This collaborative approach supports consistent application of personalised strategies and strengthens accountability for medication-related outcomes [42]. The expanding role of pharmacists reflects a shift from traditional medication management to active participation in data-driven, multidisciplinary therapeutic decision-making.

Scalability and Health System Implementation

The practical relevance of personalised pharmacotherapy is most evident in complex care environments such as tertiary hospitals, specialty clinics, and chronic disease management programmes, where individualised strategies can reduce adverse events and improve therapeutic alignment [14]. Pharmacist-led personalised services have demonstrated feasibility when supported by institutional commitment and standardised protocols [23]. However, scalability introduces challenges, including the need to balance individualisation with reproducibility to avoid fragmentation of care [38]. The integration of personalised pharmacotherapy within health systems aligns with a broader shift toward learning-based care models, where data-driven insights support continuous therapeutic optimisation [43]. This transition from reactive medication adjustments to proactive, structured medication management reflects the evolving role of clinical pharmacy in routine practice [44]. Table 3 summarises key strategies for integrating personalised pharmacotherapy into clinical pharmacy practice.

**Table 3:** Practice-level strategies for integrating personalised pharmacotherapy EHR: electronic health record; CDS: clinical decision support; PGx: pharmacogenomics; PK: pharmacokinetics; PD: pharmacodynamics

Integration Domain	Strategy	Role of Clinical Pharmacist	System-Level Requirement	Expected Impact	References
Workflow integration	Embedding decision tools into prescribing pathways	Interpretation, validation, follow-up	Interoperable EHR and CDS systems	Consistent application of personalisation	[41]
Workforce development	Advanced training in PGx, PK/PD, and data analytics	Clinical leadership and stewardship	Credentialing and continuing education	Improved clinical confidence and adoption	[31]
Interprofessional collaboration	Multidisciplinary therapy planning	Data synthesis and medication optimisation	Defined roles and shared accountability	Enhanced therapeutic alignment	[6]
Monitoring & feedback	Outcome tracking and reassessment	Performance evaluation and adjustment	Access to real-world data	Continuous improvement in care quality	[12]
Service scalability	Standardised protocols with local adaptation	Oversight of reproducibility	Institutional commitment	Sustainable personalised care models	[44]

Performance evaluation and clinical Impact of personalised pharmacotherapy

Assessment of Clinical Impact

Evaluation of individualised pharmacotherapy requires moving beyond mechanistic plausibility to demonstrate measurable clinical impact at the patient and population levels [45]. While improved prediction of drug exposure and response supports the rationale for personalised approaches, their value ultimately depends on achieving better health outcomes, reducing adverse events, and optimising resource utilisation [18]. This has led to increased emphasis on performance metrics that reflect real-world effectiveness rather than relying solely on surrogate or intermediate endpoints [26]. Comparative evaluation frameworks are increasingly used to assess personalised strategies against conventional approaches in pragmatic clinical settings [12]. Such assessments must account for variability in implementation fidelity, patient adherence, and clinical context, all of which significantly influence observed outcomes [7]. Pharmacist-led interventions have shown promise by integrating personalisation tools within structured medication management frameworks that support consistent application and follow-up [1]. However, attributing improved outcomes solely to personalisation remains challenging without well-designed studies that address confounding factors and selection bias [29].

Variability in Effectiveness and Clinical Context

The effectiveness of personalised pharmacotherapy is not uniform across all patient populations or clinical environments. Certain strategies demonstrate clear benefit in high-risk populations or in therapies with narrow therapeutic indices, whereas others may offer only marginal improvements despite increased complexity [21,30]. This heterogeneity underscores the importance of prioritising interventions based on clinical relevance and expected benefit, rather than indiscriminate implementation of emerging technologies [46]. Context-specific evaluation is therefore essential to identify where personalisation provides a meaningful clinical advantage.

Continuous Evaluation and System-Level Impact

Performance evaluation should be viewed as a continuous, iterative process that informs optimisation of therapeutic strategies and healthcare delivery systems. The use of performance data enables refinement of intervention design and prioritisation of approaches with demonstrated clinical impact [6]. Monitoring outcomes such as hospitalisation rates, treatment-related toxicity, and adherence supports ongoing optimisation of care pathways at the individual level [14]. Additionally, performance assessment functions as an accountability mechanism, contributing to continuous improvement in pharmacotherapy practices [38]. Robust evaluation frameworks are therefore essential to sustain confidence in personalised pharmacotherapy and to guide its rational integration within healthcare systems [47].

Convergence of multi-omics, modelling, and digital innovations in clinical pharmacy

Multi-omics Integration and Mechanistic Insights

Multi-omics technologies, quantitative modelling, and digital innovations represent a significant advancement in personalised pharmacotherapy, transforming therapeutic optimisation within clinical pharmacy practice [48]. Genomics, transcriptomics, proteomics, and metabolomics provide complementary insights into disease biology and drug response, enabling more comprehensive patient stratification than single-modality approaches. When integrated with pharmacokinetic-pharmacodynamic modelling, these data layers support mechanistic interpretation and facilitate individualised dosing and treatment selection [29]. However, effective implementation requires structured frameworks capable of translating complex and heterogeneous data into clinically interpretable outputs [12]. This integration is further supported by computational modelling and AI-based approaches that identify patterns within multidimensional datasets and enable dynamic assessment of patient status [37]. Importantly, the clinical application of these models must remain grounded in established pharmacological principles and validated within real-world practice settings [30]. The resulting multi-omic insights must be aligned with patient-centred treatment goals and safety considerations to support informed therapeutic decisions [6].

Clinical Applications in Complex Diseases

Integrated personalisation approaches are particularly relevant in complex chronic diseases and advanced therapeutic contexts, where conventional trial-and-error strategies are inefficient and resource-intensive [34]. Multi-omics-guided decision-making can support early identification of non-responders, optimise therapy sequencing, and reduce unnecessary treatment escalation [21]. These applications enhance therapeutic precision by enabling earlier and more targeted interventions based on multidimensional patient data.

System-Level Integration and Future Implications

Scalability of multi-omics approaches is facilitated through digital platforms that embed these insights into decision-support systems, promoting consistency while preserving patient-level individualisation [41]. This convergence reflects a transition from isolated precision tools to interconnected therapeutic ecosystems [1], positioning clinical pharmacy as a central interface for integrating biological, quantitative, and digital data streams [14]. Despite these advances, implementation remains heterogeneous, with variability in infrastructure, data integration capacity, and workforce readiness limiting widespread adoption [46]. The convergence of multi-omics, modelling, and digital technologies represents a continuum from discrete innovations to integrated clinical systems [49], supporting the development of predictive, adaptive, and patient-centred medication management frameworks within healthcare systems [50].

Limitations and Future Recommendations

Personalised pharmacotherapy remains limited by heterogeneous evidence quality, the lack of large prospective outcome studies, and inconsistent implementation across healthcare settings. Many investigations rely on surrogate endpoints or narrowly defined populations, reducing applicability to routine clinical practice. Fragmented integration of pharmacogenomics, biomarkers, and modelling approaches further constrains consistent therapeutic decision-making. Resource constraints, variability in workforce training, and unequal access to precision technologies, particularly in low- and middle-resource environments, also impede scalability. These factors complicate attribution of observed benefits specifically to personalisation strategies and hinder standardised performance assessment.

Future research should emphasise integrative, outcome-focused study designs that evaluate personalised pharmacotherapy within real-world clinical workflows. Development of interoperable decision-support platforms, standardised performance metrics, and structured training pathways focused on advanced pharmacotherapy will be critical for sustainable adoption. Greater emphasis on multi-omics integration, adaptive modelling, and learning health systems may enhance predictive accuracy and clinical responsiveness. Expanding evidence generation across diverse populations and care settings will strengthen equity and translational relevance, positioning medication management for broader implementation. Policy alignment should evolve accordingly.

## Conclusions

Personalised pharmacotherapy has progressed from a conceptual ambition to a clinically meaningful paradigm that increasingly defines modern pharmacy practice. This review demonstrates that the true promise of personalisation does not reside in isolated technologies, but in their deliberate integration across genetic, biomarker, pharmacometric, and digital domains. Mechanistic insight, quantitative modelling, and clinical expertise must function collectively to support informed, adaptive therapeutic decisions. Within this integrated framework, clinical pharmacists serve as critical interpreters and coordinators, translating complex data streams into patient-centred actions and sustaining continuous optimisation. The analysis reinforces that value is achieved not through innovation alone, but through structured systems that enable interpretation, monitoring, and learning within routine care. When effectively embedded, personalised pharmacotherapy improves therapeutic coherence, consistency, and safety, particularly in settings characterised by complexity and uncertainty. The enduring impact of personalised approaches will depend on sustained integration, professional leadership, and system-level alignment. As healthcare increasingly emphasises precision and value, clinical pharmacy is well-positioned to guide data-informed medication management that remains scalable and responsive to needs.
